# Hepatitis E Virus Genotype 3 in Colombia: Survey in Patients with Clinical Diagnosis of Viral Hepatitis

**DOI:** 10.1371/journal.pone.0148417

**Published:** 2016-02-17

**Authors:** Julio Rendon, Maria Cristina Hoyos, Diana di Filippo, Fabian Cortes-Mancera, Carolina Mantilla, Maria Mercedes Velasquez, Maria Elsy Sepulveda, Juan Carlos Restrepo, Sergio Jaramillo, Maria Patricia Arbelaez, Gonzalo Correa, Maria-Cristina Navas

**Affiliations:** 1 Grupo de Gastrohepatologia. Facultad de Medicina, Universidad de Antioquia, Medellin, Colombia; 2 Grupo de Investigación e Innovación Biomédica GI^2^B, Facultad de Ciencias Exactas y Aplicadas, Instituto Tecnologico Metropolitano, Medellin, Colombia; 3 Hospital Pablo Tobon Uribe, Medellin, Colombia; 4 Grupo de Epidemiología. Facultad Nacional de Salud Publica, Universidad de Antioquia, Medellín, Colombia; Texas A&M Health Science Center, UNITED STATES

## Abstract

**Background:**

Hepatitis E virus is a major cause of outbreaks as well as sporadic hepatitis cases worldwide. The epidemiology of this enterically transmitted infection differs between developing and developed countries. The aims of this study were to describe HEV infection in Colombian patients and to characterize the genotype.

**Methods:**

A prospective study was carried out on 40 patients aged over 15 with a clinical diagnosis of viral hepatitis, recruited from five primary health units in the city of Medellin, Colombia. Fecal samples obtained from the 40 consecutives cases were analyzed for HEV RNA using nested reverse transcription PCR for both ORF1 and ORF2-3. The amplicons were sequenced for phylogenetic analyses.

**Results:**

Nine (22.5%) cases of HEV infection were identified in the study population. Three HEV strains obtained from patients were classified as genotype 3. No significant association was found between cases of Hepatitis E and the variables *water drinking source*, *garbage collection system* and *contact with pigs*.

**Conclusions:**

This is the first prospective study of hepatitis E in Colombian patients. The circulation of the genotype 3 in this population is predictable considering the reports of the region and the identification of this genotype from pigs in the state of Antioquia, of which Medellin is the capital. Further studies are necessary to establish whether zoonotic transmission of HEV is important in Colombia.

## Introduction

Hepatitis E virus (HEV) is a waterborne agent and a major cause of hepatitis worldwide. It is estimated that 20 million HEV cases occur each year, including 3 million symptomatic cases, 56,000 fatalities and 3,000 abortions [1].

HEV is the most important etiologic agent of acute hepatitis epidemics related to contaminated drinking water in Asia and Africa [2]. Sporadic cases are associated with contaminated water and ingestion of raw or undercooked meat in developed countries but also in some developing nations [1, 3, 4].

HEV infection is usually an acute, self-limiting illness [4, 5]. However, severe hepatitis has been described in pregnant women and chronic HEV infection in immunocompromised patients [6–8]. Interestingly, some cases present extrahepatic manifestations, including neurological pathologies, impaired renal function, acute pancreatitis and hematological disorders [9].

HEV is a single-stranded, positive sense RNA virus, classified in the genus *Orthohepevirus*, family *Hepeviridae* [10]. The genome of 7.2 Kb contains three open reading frames (ORFs) and two 5’ and 3’ non-translated regions [11]. Four genotypes have been identified based on the molecular characterization of mammalian HEV sequences. Genotypes 1 and 2 are restricted to humans and frequently associated with epidemic hepatitis E. Genotypes 3 and 4 have been isolated from sporadic cases of hepatitis in humans but also identified in samples from a number of species including pigs [12–14].

In Latin America, the first report of HEV infection was documented in Mexico during outbreaks between 1986 and 1987; HEV Genotype 2 was identified from these cases [15]. However, no other outbreaks have since been described from the region; although many epidemiological variations have been reported [16], including the epidemiological pattern, seropositivity among different populations and the circulating genotype. Although HEV 3 is the predominant genotype in Central and South America [17–22], a few cases of HEV 1 infection have also been identified from Cuba, Venezuela and Uruguay [18, 22, 23]. Two recent retrospective studies of sera obtained from Colombian patients indicated anti-HEV IgG antibody prevalences of 7.5% (26/344) and 31.2% (342/1097) [24, 25].

The purpose of the present study was to carry out a prospective study of HEV infection in Colombian patients with a clinical diagnosis of viral hepatitis and to characterize the genotype involved.

## Methods

### Study population

A cross-sectional study was carried out in a cohort of 40 consecutives patients with clinical diagnosis of viral hepatitis, recruited when reporting for medical consultation in five public primary health units between April 2008 and July 2009. The units, located in the Medellin *comunas* of Santo Domingo, Santa Cruz, San Cristobal, Buenos Aires and Castilla serve low income populations from these neighborhoods. Moreover, these *comunas* showed the highest incidence of Hepatitis A virus infection in Medellin, second largest city in Colombia and capital of the state of Antioquia in the NW of the country [26].

The objectives of the project were discussed with the medical staff of these units who were then instructed to explain the study to the patients. An informed consent form was signed by each patient. In those under 18 years old, the need for informed consent was explained by a physician to each patient whose mother or father then signed it in on his/her behalf.

Patients older than 15 years old with jaundice, fever, vomiting, nausea and/or abdominal pain, presenting epidemiological characteristics consistent with a clinical diagnosis of viral hepatitis and able to provide a fecal sample were recruited to the study. Following recruitment, socio-demographic and clinical data were obtained from clinical reports and interviews of the patients carried out by one of the researchers. These socio-demographic characteristics included number of people living at home per room, type of floor, presence of domestic animals in the house (pets and pigs), source of drinking water and system of garbage collection.

Approximately 2–3 weeks later, all the patients were contacted again by phone in order to record any symptoms manifested since recruitment. Subsequently, two cases with diagnoses other than viral hepatitis were identified, one patient presenting autoimmune hepatitis and the other cholelithiasis. These two cases were excluded from the study.

### Specimen collection and testing

A fecal sample was obtained from each of the 40 patients, one or two days after the consultation. These samples were stored at -70°C until analyses could be carried out.

Total RNA was extracted from fecal samples using TRIzol^®^ reagent (Invitrogen, USA). To avoid cross-contamination, RNA extraction was carried out in sets of 10 samples under sterile procedures.

Two sets of degenerate primers targeting the regions ORF1 (12–359 nt) and ORF2-3 (5235–5398 nt) were used to amplify the viral genome [27, 28]. Reverse transcription was done using total RNA in sets of five samples using sterile filter pipette tips. Positive (pig fecal sample) and negative (sterile distilled water) controls were included in each RT-PCR assay.

The retrotranscription reaction was performed at 42°C for 50 min with SuperScript II (Invitrogen, USA) using the primers ORF1R (taccaccgctgracrtc, 343–359 nt) or HE364 (ctgggmytggtcdcgccaag, 5380–5398 nt) for ORF1 and ORF2/3, respectively.

The PCR of ORF1 was performed on 5 μL of cDNA using a final reaction mix (25 μL) containing buffer reaction 1X, MgCl_2_ 2.2 mM, dNTPs 0.2 mM, primers 2.0 μM ORF1R, ORF1F (ccaycagttyathaaggctcc, 12–32 nt) and 1.25 U Biolase^™^ DNA Polymerase (Bioline, USA) using sterile filter pipette tips. The PCR consisted of a 3 min step at 95°C, followed by 40 cycles at 94°C for 30 s, 45°C for 30 s and 72°C for 40 s and an extension step at 72°C for 5 min.

The PCR of ORF2-3 was carried out on 2 μL of cDNA in a final reaction mix of 25 μL containing buffer reaction 1X, MgCl_2_ 2.0 mM, dNTPs 0.2 mM, primers 0.2 μM HE364, HE361 (gcrgtggtttctggggtgac, 5235–5254 nt) and 1U Biolase^™^ DNA Polymerase (Bioline, USA) using sterile filter pipette tips. PCR cycling conditions consisted of a touchdown PCR: initial step 3 min at 95°C, cycling steps (x20) of denaturation at 94°C for 30 s, annealing temperature starting at 60°C for 30 s (decreasing by 0.5°C/1 cycle) and extension at 72°C for 20 s; followed by 20 cycles at 94°C for 30 s, 59°C for 30 s, 72°C for 20 s and a final extension step at 72°C for 7 min.

Nested amplification of ORF1 and ORF2-3 was performed using sterile filter pipette tips on 2 μl of the first round PCR product under the same conditions using the primers ORF1FN (ctcctggcrtyacwactgc, 29–47 nt), ORF1RN (ggrtgrttccaiarvacytc, 181–200 nt) and HE363 (gmytggtcdcgccaaghgga, 5375–5394 nt), HE366 (gytgattctcagcccttcgc, 5258–5277 nt), respectively. Four other previously published RT-PCR strategies were tested but the results were not reproducible [27, 29, 30].

The PCR products were run in 2% agarose gels stained with ethidium bromide.

Nucleotide sequences of amplicons were determined by automated dideoxy-sequencing (Macrogen, Inc. Seoul, Korea). Phylogenetic tree was constructed based on a combination of the ORF 1 (47–181 nt) and ORF 2–3 (5277–5375) sequences considering the limited size of the fragments amplified. The sequences were aligned with 47 prototype sequences of genotype 1–4 HEV available in GenBank database (National Center for Biotechnology Information, National Institutes of Health). Sequences were assembled with the Vector NTI software (Invitrogen, USA) and were aligned using the Clustal W Multiple Alignment application of BioEdit 7.0.5.3 [31]. Phylogenetic analysis was performed with the MEGA 6.06v program (Molecular Evolutionary Genetics Analysis) according to the Neighbor-Joining method with genetic distances evaluated with the Kimura 2 parameter corrections. Reliability of the trees was evaluated statistically by bootstrap analyses of 1000 replicates.

### Ethics statement

This study was approved by the Ethic Committees of the Universidad de Antioquia (N.125, SIU, Universidad de Antioquia) and the Hospital Pablo Tobon Uribe (N. 01–2008, HPTU). The informed consent procedure was approved by the respective ethics committees of the two institutions. The research adhered to conditions in the Declaration of Helsinki.

### Statistical Analysis

Statistical analysis was carried out using the Statistical Package for the Social Sciences (SPSS) version 18 and EPIDAT version 4. Significant differences between the frequencies were assessed by Chi-Square test. A *P* value <0.05 was considered to be significant.

## Results

The study population consisted of a cohort of 40 patients with a clinical diagnosis of viral hepatitis, attending five public primary health units in low socio-economic level neighborhoods of Medellin between April 2008 and July 2009. Based on the epidemiological data, these 40 cases of sporadic viral hepatitis were otherwise unrelated. Twenty-three patients were males (57.5%) and 17 were females (42.5%). None of the female patients was pregnant. The median age of patients was 27 years (interquartile ranging 18–34.5 years). The average period between symptom onset and consultation at the health unit was 6.2 days (interquartile range 3–8 days). The most important sign and symptoms of the patients were jaundice (92.5%), choluria (82.5%), fever (77.5%), abdominal pain (52.5%) and nausea (47.5%). Mean levels of serum Bilirrubin, Aspartate Transaminase and Alanine Transaminase were 6.5 ± 5.6 mg/dL, 324 ± 253 IU/L and 537.4 ± 544 IU/L, respectively. These data were obtained from 23/40 patients.

With respect to serological markers of viral hepatitis, 40% (16/40) of patients were positive for anti-Hepatitis A Virus IgM and 10% (4/40) positive for anti-HBc IgM and HBsAg. None of the samples was positive for anti-HCV.

The drinking water source for 31 patients (77.5%) was the city of Medellin’s water supply, the remaining nine (22.5%) using local piped water supplies. Despite the low socio-economic level of most of the patients (38/40, 95%), a garbage collection system was available for almost all the study population (38/40, 95%) and all but one dwelling (97.5%) had ceramic tile floors. With respect to the other socio-demographic characteristics, 6/40 patients were living in overcrowded conditions (15%) and only one patient (2.5%) reported being in contact with pigs (Table 1).

**Table 1:** Analysis of variables and HEV infection of patients with a clinical diagnosis of viral hepatitis in the city of Medellin.

Variable	Patients positive for HEV RNA n = 9	Patients negative for HEV RNA n = 31	Odds Ratio (95% Confidence Interval)	*P*-Value
**Drinking Water Source**				
Local piped water supply	1 (11.2%)	6 (0%)	1.13 (0.2–1.62)	0.5
Medellin piped water supply	8 (88.9%)	25 (80.6%)	1	
**Domestic animals**				
Pets	1 (11.2%)	7 (22%)	0.82 (0.2–1.15)	0.1
Pigs	0 (0%)	1 (3.3%)	0.77 (0.65–0.91)	0.5
**Garbage collection system**				
No	0 (0%)	2 (6.4%)	1.01 (0.9–1.3)	0.3
**Socio-economic level**				
Low	9 (100%)	29 (93.5%)	0.77 (0.4–1.3)	0.5
Medium	0 (0%)	2 (6.4%)	1	
**No. occupants**				
Overcrowded (>3 individuals by room)	1 (11.2%)	5 (16.2%)	0.9 (0.6–1.3)	0.4
Not overcrowded	8 (88.9%)	26 (83.1%)	1	
**Type of flooring**				
Mud	0 (0%)	1 (3.3%)	0.77 (0.65–0.91)	0.5
Ceramic tile	9 (100%)	30 (96.7%)	1	

The viral genome regions ORF1 and ORF 2–3 were detected in 9/40 of fecal samples. The phylogenetic tree was constructed based on a combination of the ORF 1 and ORF 2–3 sequences by the neighbor-joining method. Only three sequences were included in this analysis because of technical problems with the RT-PCR efficiency and quality of some sequences obtained in different assays. The distance matrix analysis showed that the 3 Colombian sequences are not identical and are different from the sequences included in the phylogenetic analysis (data not shown).

**Fig 1:**
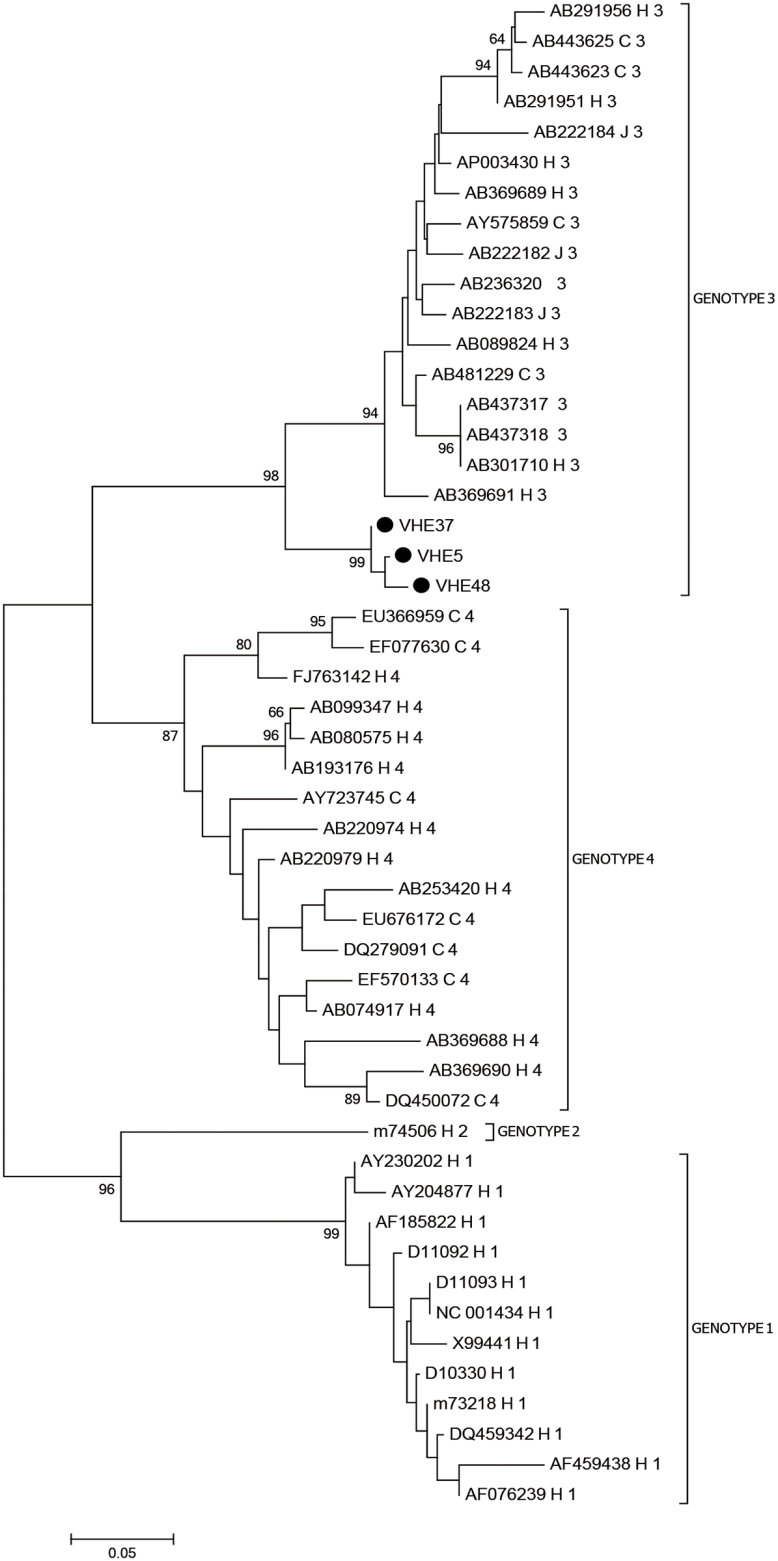
Phylogenetic analysis of HEV strains. Phylogenetic tree of partial ORF1 (47–181 nt) and ORF 2–3 (5277–5375 nt) HEV sequences derived from patients with a clinical diagnosis of viral hepatitis generated using the neighbor-joining method. The reference strains are identified with GenBank accession number, species (H: human, S: swine and WB: wild Boar), country and genotype. Colombian strains (VHE5, VHE37, VHE48) are shown with black circle. Numbers at the nodes indicate bootstrap percentages over 1000 replicates (only values > 70% are shown).

The sequences *Colombia/VHE5*, *Colombia/VHE37 and Colombia/VHE48* clustered in genotype 3 (Fig 1). The characterization of the subtype was not available considering the limited size of the HEV PCR products (ORF1 134nt, ORF2-3 98nt) obtained in this study.

HEV sequences obtained from other Latin American countries were not included in this analysis, given that the viral genome regions used for phylogenetic analysis in those studies were different from the sequences analyzed in this study. Unfortunately, the other RT-PCR strategies previously published did not yield results [27, 29, 30].

The sequence *Colombia/VHE5* was detected in a patient (female, 38 years old) with jaundice, fever, abdominal pain, vomit, nausea, hepatomegaly and choluria. Her fecal samples were obtained eight days after onset of the symptoms. The patient was negative for serological markers of Hepatitis A virus (HAV), Hepatitis B Virus (HBV) and Hepatitis C Virus (HCV). She resided in a very low socio-economic level neighborhood with no garbage collection system and obtained her drinking water from a local supply rather than that of the city of Medellin (Table 2).

**Table 2:** Demographic and clinical data for Hepatitis E Virus infection among patients with clinical diagnoses of viral hepatitis in Medellin.

Code	Sex	Age	AST IU/L	ALT IU/L	Hepatitis Viral Markers	Water supply
VHE005	F	38	ND	ND	Negative	Local
VHE013	M	42	427	865	HBsAg, anti-core IgM	Medellin
VHE023	F	17	219	418	Negative	Medellin
VHE032	F	40	ND	ND	IgM HAV	Medellin
VHE037	M	40	360	978	Negative	Medellin
VHE048	M	34	483	734	Negative	Medellin
VHE053	M	38	320	151	Negative	Medellin
VHE057	M	25	ND	ND	IgM HAV	Medellin
VHE058	M	17	203	788	IgM HAV	Medellin

The sequence *Colombia/VHE37* was detected in a patient (male, 40 years old) with jaundice, fever, choluria and a sevenfold increase of ALT and AST levels. The patient was positive for serological markers of HBV (HBsAg, anti-HBc IgM) and negative for HAV and HCV. His fecal samples were obtained nine days after onset of the symptoms. He also resided in a low socio-economic level neighborhood, although with a garbage collection system and access to drinking water from the Medellin supply (Table 2).

The sequence *Colombia/VHE48* was detected in a patient (male, 34 years old) with jaundice, fever, abdominal pain and choluria. This patient was negative for serological markers of HAV, HBV and HCV. His fecal samples were obtained eight days after the onset of the symptoms. He resided in a median socio-economic level neighborhood with a garbage collection system and access to drinking water from the Medellin supply (Table 2). None of these three patients declared having been in contact with farm animals.

HEV infection was more frequent in patients aged 34–40 (66%, 6/9) and in male patients (66%, 6/9). With regard to the other socio-demographic characteristics of the study population, the variables *local water supply*, *garbage collection system not available*, *pigs in the house* and *type of household flooring* were not significantly associated with HEV cases (Table 1).

## Discussion

The diagnosis of HEV infection is not currently included in the guidelines for the diagnosis and management of viral hepatitis used by health authorities in Colombia. However, identification of these cases is important given the risk of hepatic failure, chronic infection, neurological syndromes and renal injury; in addition, this infection could be also related to autoimmune hepatitis [32].

Although cases of Hepatitis E have never been reported in Colombia, four studies have recently been carried out on samples from different populations showing the seroprevalence of HEV infection [24, 25, 33, 34]. These studies present data of seroprevalence from pig farm workers (11.25% and 15.7% anti-HEV), people living with workers who have occupational exposure (5.9% anti-HEV) and the general population (7.2% anti-HEV) of Antioquia [33, 34]. Antioquia is the predominant region for commercial pig production farming in Colombia [35].

Further, two retrospective studies of seroprevalence have been carried out with serum samples from patients. In the first study, 8.7% of 344 samples analyzed were positive for anti-HEV (IgM/IgG). All serum samples included in this study were negative for serologic markers of HBV and HCV infection while 8.4% of samples were positive for anti-HAV IgM [24]. In the second retrospective study, 1097 samples positive for anti-HAV IgM, anti-HCV or HBsAg were selected to determine anti-HEV prevalence; 31.2% were positive for anti-HEV IgG and 11.5% for anti-HEV IgM [25].

The present study is the first prospective one of HEV infection carried out in Colombia. Forty patients aged over 15 years old with a clinical diagnosis of viral hepatitis were recruited in health centers located in five representative low socio-economic level neighborhoods of Medellin. Nine cases of HEV infection were identified in this study, constituting 22.5% of the study population. Diagnosis of hepatitis E was based on HEV-RNA positivity in fecal samples as well as clinical and epidemiological data of the patients.

Presence of viral RNA in stools could provide a good marker of HEV infection in the first days of infection, as in the present study where the average period between symptom onset and consultation was 6.2 days (interquartile range 3–8 days), particularly given the variable performance of the commercial ELISA tests available [8, 9, 36]. Indeed, in sporadic acute hepatitis E and in cases occurring during an outbreak fecal shedding of virus has been demonstrated within a week of the onset of symptoms and up to one month afterwards [36, 37].

None of the socio-demographic variables was associated with hepatitis E cases in the study population. In other studies carried out in Latin America, HEV seropositivity has been associated with consumption of untreated water [38]. In the present study, all patients had access to piped drinking water from Medellin or local (San Cristobal) supplies.

Dual infection with HEV and HAV was identified in 3/9 patients. Simultaneous infection of these enterically transmitted viruses resulting in acute viral hepatitis has already been described in patients from developing countries such as Cuba (12.8%), India (11.5%) and Egypt (4.5%) [39–41].

One patient was positive for HEV RNA and the acute infection markers of HBV. Although this coinfection is less common, a study of 162 children with sporadic acute hepatitis in Egypt and a recent study carried out with 1097 serum samples from patients in Colombia did find both hepatotropic viruses together [25, 39].

The viral genome (ORF 1 and ORF 2/3) was amplified in 22.5% (9/40) of the fecal samples obtained from the patients. A phylogenetic tree was constructed based on ORF 1 and ORF 2–3 sequences and genotype 3 was identified in the samples. The report of HEV genotype 3 in Colombia is in agreement with the results of a recent study carried out on pig farms in Antioquia [42, 43]. Unfortunately, the data available for the HEV strains obtained from patients in this study and those identified in pigs is too incomplete to establish a genetic relationship and support the hypothesis of zoonotic transmission. Further studies are necessary to demonstrate whether the HEV strains circulating among pigs and humans are closely related, particularly in Colombian states such as Antioquia where there is extensive commercial pig production [35].

The circulation of this genotype in Colombia is to be expected, given the numerous reports of HEV genotype 3 in patients and pigs from other countries of the region. HEV 3 is the currently the most important genotype in the Americas [18–22, 44–47].

The first study of HEV in pigs in the region was carried out in Argentina [48]. HEV genotype 3 strains obtained from piglets were found to be closely related to strains isolated from patients with hepatitis E cases from that country [46]. Another study carried out in Brazil confirmed the close relationship between the HEV strains circulating in pigs and in humans [47, 49]. Several reports of HEV infection in Mexico [50, 51], Chile [52], Costa Rica [53] and Bolivia [21] also demonstrated the circulation of HEV genotype 3 in pigs.

Despite the fact that there have been several reports of HEV infection in human and pigs in the region, there is currently insufficient evidence for zoonotic transmission in some countries of South America. Indeed, the subtypes of HEV genotype 3, 3b, 3c and 3f, have been characterized in strains from pigs in Brazil [17, 49, 54, 55] and 3i from Bolivia [21, 56] while circulation of HEV subtypes 3a, 3b, 3e and 3h has been reported in patients in Argentina, Bolivia and Uruguay [18, 20, 21, 45, 56]. However, identification of subtypes 3f, 3c and 3e in both human and pig populations and the close relationship among HEV strains in France demonstrated zoonotic transmission of HEV through the consumption of raw or undercooked pork and pig products [9, 57].

Some limitations of the present study have to be considered. Cases were diagnosed by medical doctors during consultations based on symptoms and epidemiological patterns but before obtaining AST, ALT and Bilirrubin serum level data. Moreover, these data were obtained from only 23 of the 40 patients. After notification of viral hepatitis at the five health units, sampling was carried out over a period of one year in the expectation of obtaining approximately 100 samples; however, availability of fecal samples from patients reduced the sample size to 40. Finally, technical problems and the size of the fragments amplified did not allow the analysis to include HEV strains from countries of the region.

In conclusion, this prospective study of sporadic acute hepatitis cases is the first one among Colombian patients to provide data on viral genome detection in fecal samples and the characterization of genotype 3. The socio-demographic variables selected were not associated with hepatitis E cases in this population. Circulation of this genotype in Colombia is predictable based on existing epidemiological reports from the region and the identification of this genotype in pigs in Antioquia. The current impact of HEV infection and risk of zoonotic transmission in Colombia deserves further investigation at the national level given the importance of commercial pig production in this country.
